# Effect of surgery and radiotherapy on complete blood count, lymphocyte subsets and inflammatory response in patients with advanced oral cancer

**DOI:** 10.1186/s12885-018-4136-9

**Published:** 2018-03-01

**Authors:** Tadej Dovšak, Alojz Ihan, Vojko Didanovič, Andrej Kansky, Miha Verdenik, Nataša Ihan Hren

**Affiliations:** 1 Clinical Department of Maxillofacial and Oral Surgery, |University Medical Center, Ljubljana, Slovenia; 2 Department of Maxillofacial and Oral Surgery, Faculty of Medicine, University of Ljubljana, Vrazov trg 2, 1104 Ljubljana, Slovenia; 3 Institute of Microbiology and Immunology, Faculty of Medicine, University of Ljubljana, Ljubljana, Slovenia

**Keywords:** Oral cancer, Surgery, Radiotherapy, Lymphopenia, Treatment outcome

## Abstract

**Background:**

The immune system has a known role in the aetiology, progression and final treatment outcome of oral squamous cell cancers. The aim of this study was to evaluate the influence of radical surgery and radiotherapy on advanced oral squamous cell carcinoma blood counts, lymphocyte subsets and levels of acute inflammatory response markers.

**Methods:**

Blood samples were obtained from 56 patients 5 days before and 10 days after surgery, 30 days and 1 year after radiotherapy. The whole blood count, lymphocyte subsets and inflammatory response markers (C-reactive protein, erythrocyte sedimentation rate, leukocyte count, expression of index CD64 and index CD163 on neutrophils and monocytes) were measured, statistically analysed and correlated with clinical treatment outcomes.

**Results:**

The post-operative period was characterised by the onset of anaemia, thrombocytosis, lymphopenia with reduced B lymphocyte, T helper cell and NK cell counts, and a rise in acute phase reactants. Immediately after radiotherapy, the anaemia improved, the lymphopenia worsened, and thrombocyte levels returned to pre-treatment values. There was a drop in counts across the T and B cell lines, including a reduction in B lymphocytes, naïve and memory T cells with reduced CD4+ and CD8+ counts and a decreased CD4/CD8 ratio. One year after radiotherapy all the lymphocyte subsets remained depressed, the only exception being NK cells, whose levels returned to pre-treatment values.

**Conclusions:**

We concluded that surgery resulted in a stronger acute phase response than radiotherapy, while radiotherapy caused a long-lasting reduction in lymphocyte counts. There was no correlation between any of the pre-treatment parameters and the clinical outcome.

## Background

Oral and pharyngeal cancer is the 7th most common cancer and the 9th most lethal in the European Union [1]. The treatment for early-stage oral squamous cell cancers (OSCC) is generally single modality, either surgery or radiotherapy. Treatment of early-stage oral squamous cell cancers (OSCC) is generally unimodal using either surgery or radiotherapy (RT). The treatment for locoregionally advanced OSCC is multimodal, with either surgery followed by adjuvant radiation or chemoradiation (as indicated by pathologic features), or definitive chemoradiation. Survival of patients with oral cancer mainly depends on the stage of disease. More than 50% of patients with oral cancer have advanced disease at the time of diagnosis [2]. By introducing new cytotoxic therapies to patients’ treatment regimens, the survival rates have improved in the last decade [3].

Lymphocytes are involved in most human immune system mechanisms aimed at identifying and removing cancer cells [4]. Patients with cancer are known to have abnormalities in T-cell and B-cell counts [5].

The microenvironment of head and neck squamous cell carcinomas (HNSCC), which include OSCC amongst them, is characterized by an imbalanced cytokine profile, favouring immunosuppressive over stimulatory cytokines [6]. It is also known that immunodeficiency increases HNSCC risk [7]. Infiltration of regulatory T cells into the tumour microenvironment was shown to promote tumour-induced immune modulation and subsequent tumour progression [8].

Patients with HNSCC have reduced levels of CD3+, CD4+, and CD8+ T cells in peripheral blood and CD4+ levels show correlation with the stage of disease [9].

Injury from surgical trauma follows dynamic pattern and results in haemodynamic, metabolic and immune changes in patients in the postoperative period. The initial systemic inflammatory response is mediated primarily by the cells of the innate immune system. Once healing of the injured site has begun, an anti-inflammatory response becomes prominent. This anti-inflammatory or immunosuppressive phenotype is mediated primarily by the cells of the adaptive immune system. After major surgery, the functions of innate and of cell- mediated immunity are dramatically paralyzed [10].

Radiotherapy is one of three treatment modalities in oral cancer patients. Depending on the site and dose of radiation majority of patients will experience signs of acute toxicity that are self-limited in duration [11]. Lymphocytes are sensitive to radiation and radiation of areas rich in lymphatics and large vessels produces significant and long lasting immune alterations [12].

The aim of our prospective non-randomized study was to evaluate the effect of major surgical procedures and RT on the complete blood count, lymphocyte subpopulations and acute inflammatory response markers in the peripheral blood of patients with advanced oral squamous cell carcinoma (AOSCC). Partial preliminary results have already been published [13].

## Methods

Patients were selected from the prospective non-randomized study, running from 2008 to 2013 on the Clinical Department for Oral and Maxillofacial Surgery, University Clinical Center in Ljubljana, according to the following inclusion criteria:

- Histologically verified squamous cell carcinoma of the oral cavity Stage III and IV according to the American Joint Committee on Cancer 2010 staging.
- Surgery and radiotherapy as the only treatment modalities.
- Patients’ first malignant tumour.
- No prior radiotherapy.
- Completed blood sampling prior to surgery, after surgery, after radiotherapy, 1 year after the radiotherapy.

The study protocol was approved by the Republic of Slovenia National Medical Ethics Committee (No. 79/06/07; 19 April 2007), and an adequate written consent was obtained from each patient. Protocol of the study did not affect the standard treatment protocol of the patients in any way. The primary treatment modality of oral cancer is generally determined by the stage of the disease, with surgical treatment remaining the mainstay of multimodal treatment. In treatment selection our multidisciplinary board follows the national guidelines, which were adopted from the National Comprehensive Cancer Network (NCCN).

Seventy two patients were enrolled in the study in line with the inclusion criteria. At the end of the study 10 patients, who had been clinically assigned to advanced cancer, were down staged based on the histopathological report, 4 patients did not have complete blood samples and 2 patients no longer wanted to participate in the study. Of the remaining 56 patients, there were 44 men and 12 women with median age of 68 years (range, 47-89 years). Additional examinations required for the purpose of our study were blood sampling 5 days before surgery (T1), 10 days after surgery (T2), 30 days after radiotherapy (T3) and 1 year after radiotherapy (T4). The treatment outcome was evaluated 2 years after the radiotherapy. Main characteristics of patients are presented in Table 1.

**Table 1:** The main patient characteristics together with AOC stages, localizations of tumours, type of neck dissection, reconstruction type and blood sampling times

Number of patients	56		
Gender	♂ 44	♀ 12	
Age (mean (range))	62 (42-84)		
Cancer Stage AJCC2010	III (12)		
	IVA (44)		
Tumour location	Tongue	22	
	Floor of mouth	17	
	Retromolar trigonum	6	
	Gum Maxilla	5	
	Gum Mandible	6	
Neck dissection	Unilateral	25	
	Bilateral	31	
Reconstruction	None		5
	Local flap	Tongue	2
	Distal flap	Temporalis	2
		PMMF	6
	Free flap	RFF	22
		ALT	8
		Iliac crest	3
		Fibula	6
		LMF	2
Blood samples	T1- 5 days before surgery		
	T2- 10 days after surgery		
	T3- 30 days after radiotherapy		
	T4- 1 year after radiotherapy		

*PMMF* Pectoralis Major Muscular Flap, *RFF* Radial Forearm Flap, *LMF* Latissimus Myocutaneous Flap, *ALT* Anterior Lateral Thigh, *T1* blood sample before surgery, *T2* blood sample after surgery, *T3* blood sample before radiotherapy, *T4* blood sample 1 year after radiotherapy

Flow cytometry: The samples (100 μl of blood) were incubated with 10 μl of the appropriate MoAb. Antibodies against the following cell surface structures were applied: CD3, CD4, CD8, CD19, HLA-DR, CD56, CD45RA+, CD45RO, CD95 (Exalpha Biologicals, Boston, MA, USA. Non-specific isotype mouse MoAb were used as negative controls. Cells were analyzed on FACSCantoII™ Flow Cytometer (BDBiosciences), equipped with blue (488-nm solid-state) and red (633-nm helium-neon) laser. Digital data was acquired with FACSDiva software (BDBiosciences) and analyzed using FlowJo software (Tree Star Inc.).

Neutrophil CD64 expression was measured with a diagnostic kit Leuko64™ following the manufacturer’s instructions. Additionally, we used the Leuko64™ QuantiCALC automated software (Trillium Diagnostic) that reports neutrophil expression of CD64 as an index using fluorescein-labelled calibration beads. An internal negative control of the assay was provided by the automated measurement of the lymphocyte CD64 index, which had to be less than 1.0, and an internal positive control of the assay was provided by automated measurement of the monocyte CD64 index, which had to be more than 3.0. Isotype-control antibodies were routinely used in each experiment to detect a non-specific staining.

Data were presented as the average and 95% confidence interval for the average. Comparison between groups at different time intervals was performed using the Student’s t- test.

One year after last blood sampling (T4), patients were divided into two groups: the first one with no evidence of disease or with death of other cause, and the second with local, locoregional or distal failure and death of the disease. We checked for possible prognostic factors in the immune state of the patients, prior to the performance of a Student’s t-test between these two groups.

The differences were considered to be statistically significant at the level of *p* < 0.05. Statistical analysis was performed using Statistical Package for the Social Sciences for Windows, version 12.0 (SPSS Inc., Chicago, USA).

## Results

Of 56 patients that matched the inclusion criteria, 37 were free of disease at final check-up (1 year after last blood sampling - T4). Of the remaining 19, 4 patients died of other causes during the time of our study, 9 patients died of disease-related complications, 4 patients developed local progression of the disease, 1 patient developed distant metastases, and 1 patient developed a secondary tumour. Thirty seven patients that were free of disease and 4 patients that died of other causes constituted the “success” group, while the remaining 15 patients constituted the “failure” group.

Blood samples prior to surgery (T1) were collected between 5 to 13 days before surgery (mean 8 ± 2). All patients were surgically treated with en-bloc excision of tumour and modified neck dissection (31 of them bilateral), and a subsequent reconstruction (41 with free flaps and 10 with pedicled flaps). In only 5 cases it was possible to close the defect primarily. The average blood loss during surgery was 470 ml (range 250 - 1200 ml) as assessed by the anaesthesiologist and the surgeon. All except one patient were tracheotomised at the time of surgery, with 3 patients subsequently remaining tube dependent, and the remainder having the tracheostomy tube removed between 2 to 22 days after the surgical procedure (mean 8 ± 5).

Blood samples prior to radiotherapy (T2) were taken between 8 and 11 days after surgery (mean 8 ± 2). Patients were irradiated with an external beam by a 6 MV linear accelerator. They received a dose between 60 and 66 Gy (mean 62 ± 2), divided over 2 Gy daily fractions, five times a week. No patient received hyperfractionated RT or chemotherapy. RT was performed within 28 to 54 days after surgery (mean 34 ± 7). The lower border of the irradiation field was always two centimetres above the clavicle, to avoid irradiation of the thymus.

The period of time between the last dose of RT and the next blood sample collection (T3) ranged from 24 to 44 days (mean 36 ± 6). The last blood sample (T4) was collected between 206 and 517 days after radiotherapy (mean 398 ± 57).

The mean values of measured parameters at all blood sampling times are presented in Table 2 together with their standard deviations and normal reference values for peripheral blood of our hospital laboratory.

**Table 2:** Mean values and standard deviations of all measured parameters in blood samples in observed times (T1, T2, T3, T4) together with normal values

	T1 (mean ± SD)	T2 (mean ± SD)	T3 (mean ± SD)	T4 (mean ± SD)	Normal values
Erci (10^*^12/L)	4.32 ± 0.52	3.75 ± 0.48	4.45 ± 0.62	4.33 ± 0.44	4.20- 6.30
Hb (g/L)	139.2 ± 12.6	114.6 ± 17.9	134.9 ± 16.7	135.3 ± 12.2	120-180
Leu. (10^*^9/L)	8.53 ± 2.81	9.34 ± 3.06	6.9 ± 2.37	7.78 ± 5.39	4.0- 10.0
Lym (10^*^9/L)	1.9 ± 0.62	1.7 ± 0.62	1.1 ± 0.49	1.24 ± 0.64	1.4- 3.3
Pt (10^*^9/L)	278.7 ± 83.5	511.6 ± 202.2	270.7 ± 76.3	278.2 ± 92.1	140- 340
Neutr (10/9/l)	5.50 ± 2.26	6.56 ± 2.79	4.99 ± 2.12	5.54 ± 3.99	1.6- 7.5
CRP (mg/L)	9.1 ± 12.2	33.8 ± 37.4	13.3 ± 28.6	11.9 ± 23.8	< 5
ESR (mm/h)	32.1 ± 20.9	59.8 ± 21.2	39.8 ± 25.3	28.2 ± 19.6	< 15
Albumin (g/L)	42.7 ± 3.9	39.1 ± 4.2	43.9 ± 3.9	45.0 ± 3.3	32- 55
iCD64 mono.	7.5 ± 2.5	9.1 ± 3.9	11.0 ± 20.6	8.0 ± 2.9	4.34–8.70
iCD64 nevt.	0.73 ± 0.22	1.09 ± 1.46	0.93 ± 0.58	0.75 ± 0.32	0.45–2.16
iCD163 mono.	8586 ± 4129	8538 ± 4885	8667 ± 5288	8372 ± 6002	1061- 2740
iCD163 nevt.	223.4 ± 109.6	238.5 ± 153.8	252.9 ± 173.3	253.7 ± 172.9	301-435
HLA/DR3 (10^*^9/L)	0.26 ± 0.19	0.27 ± 0.25	0.28 ± 0.25	0.21 ± 0.21	0.06-0.30
CD3+ (10^*^9/L)	1.44 ± 0.52	1.32 ± 0.55	0.79 ± 0.41	0.81 ± 0.56	1.00-2.20
CD19+ (10^*^9/L)	0.17 ± 0.10	0.13 ± 0.07	0.06 ± 0.04	0.10 ± 0.06	0.11-0.57
CD4+ (10^*^9/L)	0.92 ± 0.32	0.82 ± 0.32	0.37 ± 0.17	0.38 ± 0.22	0.53-1.30
CD8+ (10^*^9/L)	0.53 ± 0.28	0.50 ± 0.32	0.41 ± 0.29	0.43 ± 0.38	0.33-0.92
CD4/CD8	2.10 ± 1.14	2.12 ± 1.27	1.11 ± 0.64	1.11 ± 0.59	1.0- 2.0
NK (10^*^9/L)	0.30 ± 0.16	0.24 ± 0.12	0.26 ± 0.14	0.34 ± 0.18	0.07-0.48
CD45RA + CD4+	0.27 ± 0.18	0.22 ± 0.13	0.06 ± 0.04	0.07 ± 0.05	0.23-0.77
CD45RO + CD4+	0.65 ± 0.25	0.59 ± 0.26	0.31 ± 0.15	0.31 ± 0.20	0.24 – 0.70

Legend: *Leu* leukocytes, *Lym* lymphocytes, *CD3+* T lymphocytes, *CD19+* B lymphocytes, *CD4+* T helper cells, *CD8+* cytotoxic T cells, *HLA/DR3* activated T lymphocytes, *NK* natural killer cells, *iCD64 mono* index of CD64 expression on monocytes, *iCD64 neutr* index of CD64 expression on neutrophils, *iCD163 mono* index of CD163 expression on monocytes, *iCD163 neutr* index of CD163 expression on neutrophils, *CD45 + RA + CD4+* naïve helper T lymphocytes, *CD45 + RO + CD4+* memory helper T lymphocytes, *CD95 + CD4+* apoptosis destined T helper cells, *Erci* erythrocytes, *Hb* haemoglobin, *Pt* platelets, *Neutr* neutrophils, *ESR* erythrocyte sedimentation rate; *CRP* C-reactive protein

Surgery resulted in anaemia, leucocytosis, lymphopenia, thrombocytosis, rise in neutrophils, CRP, erythrocyte sedimentation rate (ESR) and a reduction in albumin levels. In lymphocyte subpopulations the reductions in B lymphocyte, T helper cell, NK cell and naïve helper T lymphocyte counts were statistically significant. Out of the measured indexes, the only statistically significant increase was in the level of index CD64 on monocytes.

Radiotherapy caused a marked reduction in total lymphocyte counts, the levels of T, B lymphocytes, T helper lymphocytes, and cytotoxic T lymphocytes. CD4+ lymphocytes levels changed more than CD8+ levels, thereby also decreasing the CD4/CD8 ratio. Neither the CD64 index nor CD163 index showed any statistically significant changes during the observational period. The levels of NK cells did not change after radiotherapy and after 1 year rose to levels that were just above the pre-treatment values. CD45RA + CD4+ (naïve) T lymphocytes levels were diminished to 20% of the starting value and their value remained unchanged even after 1 year. CD45RO + CD4+ (memory) T lymphocyte levels were halved and did not recover after 1 year. After 1 year, there was still a statistically significant reduction, compared to pre-treatment values, in the levels of haemoglobin, lymphocytes, activated T lymphocytes, T lymphocytes, B lymphocytes, CD8+ and CD4+ lymphocytes, while the CD4/CD8 ratio was essentially half of that measured at T1.

The results of T-test comparing values of observed parameters at different blood sampling types are presented in Table 3.

**Table 3:** Comparison of the observed parameters between T1/T2, T2/T3, T1/T3, T3/T4, T1/T4, T2/T4 blood samples as *p* values of the t- test between compared parameters at stated blood sampling times. Statistical significant differences according to the compared value (*p* ≤ 0.05) are marked by ^*^ and (*p* ≤ 0.005) with ^**^

	T1/T2	T2/T3	T1/T3	T3/T4	T1/T4	T2/T4
Erci (10^*^12/L)	0.000^**^	0.000^**^	0.064	0.043^*^	0.719	0.000^**^
Hb (g/L)	0.000^**^	0.000^**^	0.042^*^	0.967	0.028^*^	0.000^**^
Leu. (10^*^9/L)	0.128	0.000^**^	0.001^**^	0.313	0.442	0.086
Lym (10^*^9/L)	0.011^*^	0.000^**^	0.000^**^	0.040^*^	0.000^**^	0.000^**^
Pt (10^*^9/L)	0.000^**^	0.000^**^	0.501	0.683	0.784	0.000^**^
Neutr (10/9/l)	0.024^*^	0.000^**^	0.192	0.443	0.887	0.131
CRP (mg/L)	0.000^**^	0.004^**^	0.223	0.707	0.466	0.001^**^
ESR (mm/h)	0.000^**^	0.000^**^	0.008^*^	0.000^**^	0.273	0.000^**^
Albumin (g/L)	0.000^**^	0.000^**^	0.049^*^	0.050	0.000^**^	0.000^**^
iCD64 mono.	0.011^*^	0.496	0.241	0.286	0.185	0.121
iCD64 nevt.	0.090	0.385	0.021^*^	0.008^*^	0.500	0.111
iCD163 mono.	0.915	0.861	0.979	0.931	0.555	0.585
iCD163 nevt.	0.378	0.618	0.164	0.725	0.297	0.683
HLA/DR3 (10^*^9/L)	0.989	0.534	0.436	0.002^**^	0.04^*^	0.122
CD3+ (10^*^9/L)	0.060	0.000^**^	0.000^**^	0.605	0.000^**^	0.000^**^
CD19+ (10^*^9/L)	0.006^*^	0.000^**^	0.000^**^	0.000^**^	0.000^**^	0.008^*^
CD4+ (10^*^9/L)	0.023^*^	0.000^**^	0.000^**^	0.347	0.000^**^	0.000^**^
CD8+ (10^*^9/L)	0.189	0.045^*^	0.002^**^	0.762	0.061	0.225
CD4/CD8	0.576	0.000^**^	0.000^**^	0.140	0.000^**^	0.000^**^
NK (10^*^9/L)	0.008^*^	0.592	0.050	0.001^**^	0.125	0.000^**^
CD45RA + CD4+	0.003^**^	0.000^**^	0.000^**^	0.005^*^	0.000^**^	0.000^**^
CD45RO + CD4+	0.045	0.000^**^	0.000^**^	0.646	0.000^**^	0.000^**^

Legend: *Leu* leukocytes, *Lym* lymphocytes, *CD3+* T lymphocytes, *CD19+* B lymphocytes; *CD4+* T helper cells, *CD8+* cytotoxic T cells, *HLA/DR3* activated T lymphocytes, *NK* natural killer cells, *iCD64 mono* index of CD64 expression on monocytesm, *iCD64 neutr* index of CD64 expression on neutrophils, *iCD163 mono* index of CD163 expression on monocytes, *iCD163 neutr* index of CD163 expression on neutrophils, *CD45 + RA + CD4+* naïve helper T lymphocytes, *CD45 + RO + CD4+* memory helper T lymphocytes, *CD95 + CD4+* apoptosis destined T helper cells, *Erci* erythrocytes, *Hb* haemoglobin, *Pt* platelets, *Neutr* neutrophils, *ESR* erythrocyte sedimentation rate, *CRP* C-reactive protein

One year after last blood sampling, at final check-up, we statistically correlated the measured parameters between the “success” group (no evidence of disease and death of other cause) and the “failure” group (locoregional, distant failure, secondary tumour and death of disease) at T1 for possible prognostic markers. Table 4 summarizes statistical analysis of selected parameters between both groups.

**Table 4:** is showing *p* values of the t- test at T1 between the “success” (*N* = 41) and the “failure” (*N* = 15) group of patients. Statistical significant differences according to the compared value (*p* ≤ 0.05) are marked by ^*^ and (*p* ≤ 0.005) with ^**^

Marker	Success group	Failure Group	Significany	Normal values
CRP (mg/L)	8.5	11.0	0,496	< 5
Neutr/Lym	2.9	3.3	0,414	
Pt (10^*^9/L)	277	282	0,844	140-340
CD3+ (10^*^9/L)	1.50	1.25	0,111	1.00-2.20
CD19+ (10^*^9/L)	0.17	0.16	0,717	0.11-0.57
CD45RA + CD4+	0.29	0.22	0,263	
CD45RO + CD4+	0.66	0.60	0,391	
Pt/Lym	153	173	0.363	

Legend: *CRP* C-reactive protein*, Neutr* neutrophils, *Lym* lymphocytes, *CD3+* T lymphocytes, *CD19+* B lymphocytes, *CD45 + RA + CD4+* naïve helper T lymphocytes, *CD45 + RO + CD4+* memory helper T lymphocytes, *Pt* platelets

**Fig. 1:**
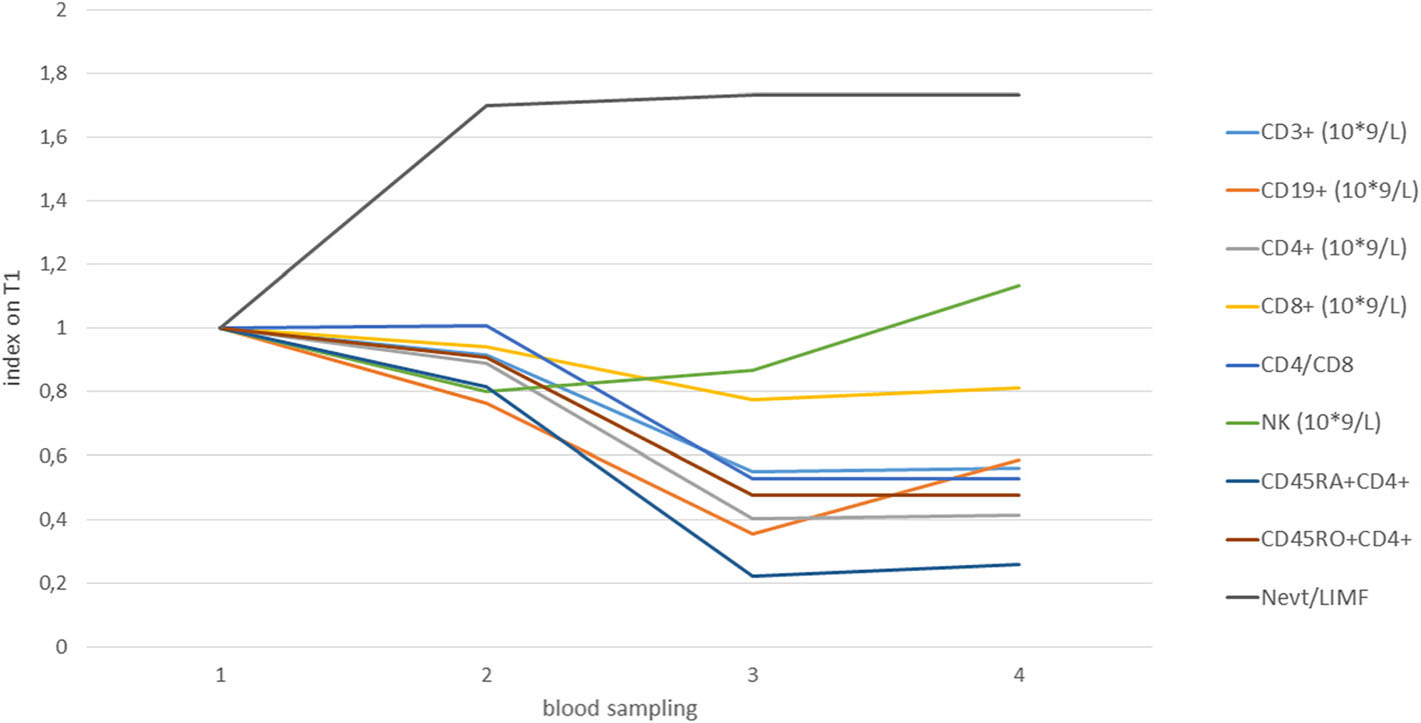
Diagram of impact of surgery and radiotherapy on lymphocyte populations (CD3+, CD19+, NK cells, CD4+, CD8+, CD45RA+, CD45RO+) indexed on T1 values

Levels of measured lymphocyte subpopulations indexed on T1 levels are shown in Fig. 1.

**Fig. 2:**
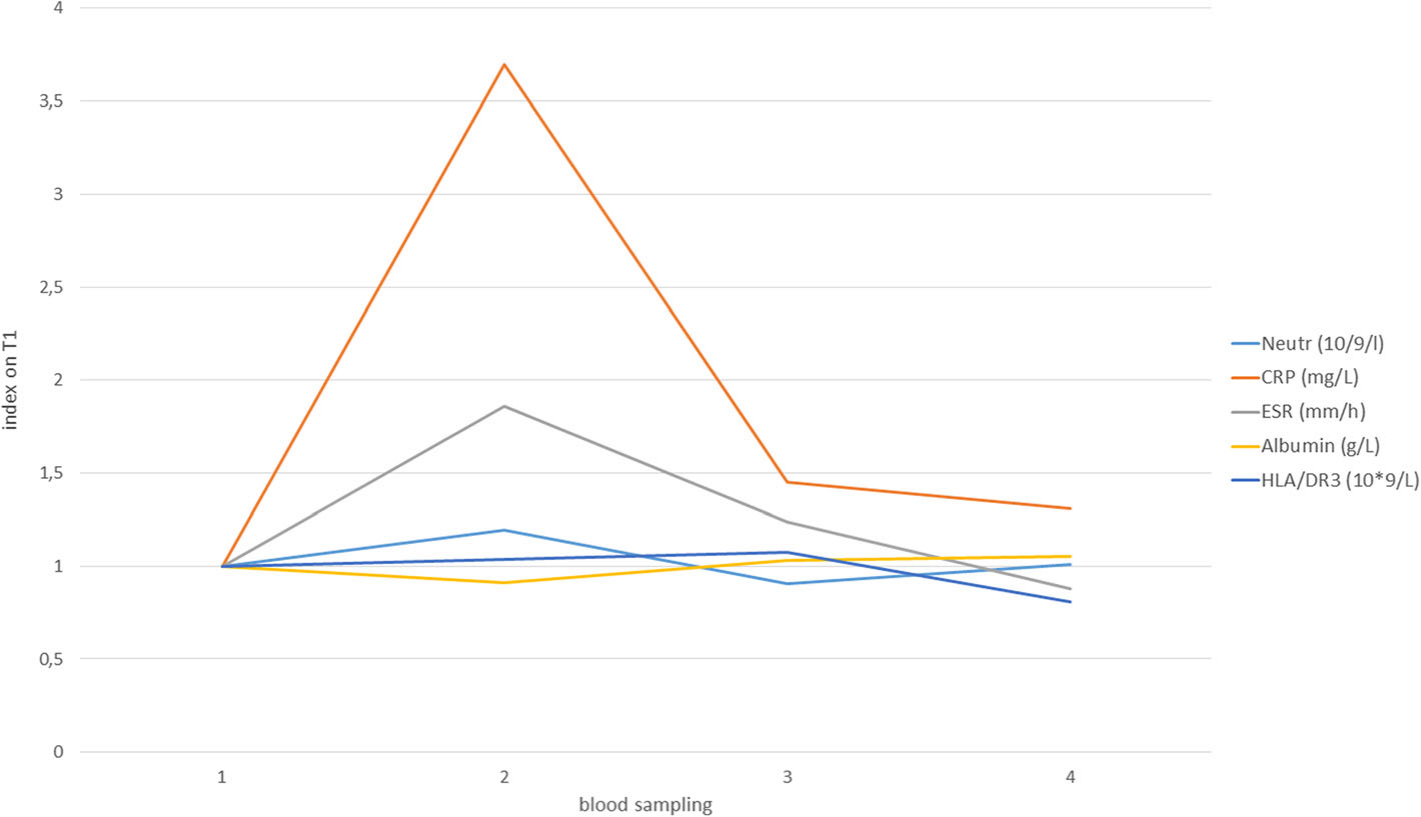
Values of HLA/DR3, ESR, Neutrophyls, Albumin and CRP indexed to T1 levels

Figure 2 shows the levels of acute phase proteins (ESR, Albumin, CRP), neutrophyls and marker of activated T helpers (HLA/DR3) at blood sampling times indexed to T1 values.

## Discussion

Radical surgical removal of the tumour is commonly the first and most important step toward the elimination of the disease. Nonetheless, during the perioperative period in oncological surgery, there might be some shedding of malignant cells, increased proliferation of tumour cells, excess release of pro-angiogenic and/or pro-invasive factors, abundant release of growth factors, psychological distress, and suppression of cell mediated immunity, which may act in synergy to render the patient temporarily vulnerable to metastases, which could otherwise have been controlled [14].

Free tissue transfer represents one of the most popular and reliable techniques for reconstruction after large primary tumours resection [15]. In only 5 of our patients it was possible to close the defect primarily or with a local flap, all others needed distal or free flap reconstruction. During such major surgery substantial blood loss may occur, varying between 500 and 1500 ml [16]. Anaemia is expected after major surgery of the head and neck, however, moderate anaemia also developed in our patients despite the loss of small volumes of blood. Hypoxia because of anaemia can decrease radiation efficacy [17]. The level of haemoglobin in our study was just below the proposed level of Hb before RT, which is 120 g/l, although in our study blood samples were taken within 8 and 12 days after surgery, thus the expected spontaneous rise prior to radiotherapy probably did occur. Microvascular reconstruction leads to prolonged operative times, and major, prolonged, surgical procedures are known to cause immunosuppression [18].

Inflammatory proteins (ESR, CRP) are non-specific markers related to infection, injury and neoplasia. They have been also correlated to cancer progression and prognosis in different malignancies [19], which was not the case in our study. In our patients, the ESR was elevated above normal before commencement of any treatment, and doubled after surgery (T2). At the T3 blood sampling the ESR remained above the initial value but with a gradual reduction towards pre-treatment values. It is of interest that ESR at T4 fell below the starting values, although the reduction failed to reach statistical significance. C-reactive protein (CRP) is an acute phase protein synthesized in the hepatocytes. CRP levels rise approximately 4 to 12 h after surgery, and peak at 24 to 72 h [20]. Once the tissue injury is resolved, CRP levels fall rapidly due to a short half-life of 6 h. Khandavilli et al. reported that increased preoperative CRP was associated with worse overall survival in patients with oral cancer [21], but a study by Krusse et al. failed to prove any correlation between preoperative CRP levels and disease progression in head and neck cancer [22]. Our results failed to show any correlation between the preoperative levels of the CRP and disease outcome. Surgery caused a 4-fold increase in CRP levels, while at T3 the levels of CRP showed a trend of returning to the pre-treatment values, although still at more than double the normal values. This is in accordance with the study by Mohammed et al. and Cheethana et al., who used ESR and CRP as biomarkers of radiation induced mucositis [23, 24]. After 1 year the CRP levels were practically identical to the preoperative values, but still above normal, which could indicate an ever-present subtle inflammation in the radiation-damaged mucosa.

There are several reports regarding the potential utility of CD64 (Fc receptor on monocytes, neutrophils, macrophages and eosinophils) on neutrophils for the diagnostic assessment of infection and systemic inflammatory response syndrome (SIRS) in adults [25].

CD163 is a monocyte/macrophage-associated antigen, which possesses anti-inflammatory properties and has an immunoregulatory role [26]. Since both indexes are only increased for short periods of a few days [27], this might explain why we failed to find any differences in the index values at various sampling times.

Various early studies have shown that cancer patients have depressed cell-mediated immune functions [28, 29]. Although we did not use a control group to match the T1 levels with healthy volunteers, it is worth noting that the average values of our measured parameters did not fall outside of reference values used by our hospital laboratory.

Leaver et al. and Ogawa et al. reported a fall in NK cells and T lymphocytes after surgery, and the levels were reduced up to 14 days after surgery [18, 30]. Additionally, the study conducted by Kuss et al. showed a persistent reduction in CD4+ cell counts in patients with a history head and neck cancer which was discovered long after surgery [29]. In our study the levels of NK cells and CD4+ cells also fell, but in contrast with the abovementioned studies, we observed a reduction in B lymphocyte levels. There was a trend towards reduced levels of T lymphocytes, but no other measured immunological parameter reached statistical significance. Although our T2 blood samples were taken within 8 and 12 days of surgery (mean 8 ± 2), one of the reasons for this discrepancy might be the small sample group.

In our group of patients radiotherapy was delivered between 28 to 54 days after surgery (mean 34 ± 7), and it only exceeded the prescribed 6 weeks’ time in 8 patients (15%), providing the appropriate treatment regimen to most of the patients in the recommended time [31].

In our study we observed that even after more than a year, post-radiotherapy levels of lymphocytes, B lymphocytes, T lymphocytes, CD4+ and CD8+ lymphocytes remained significantly diminished compared to their pre-treatment levels. A study by Verastegui et al. [12] showed that CD4+ lymphocytes have a tendency to recover better than CD8+ cells, however, we did not see the expected trend in our study. In contrast, the levels of NK cells after a year were higher (although the increase was not statistically significant) than pre-treatment levels, indicating a probable compensatory increase. The levels of B lymphocytes in the study of Verastegui et al. [12] returned to normal after 1 year, which was not the case in our study, where they remained significantly reduced even more than a year after radiotherapy.

The large amount of accumulated evidence indicates that CD4+ T cells have a pivotal role in generating and maintaining anti-tumour immune responses through their interactions with cytotoxic T lymphocytes, B lymphocytes, macrophages and NK cells [32]. The levels of CD4+ lymphocytes more than halved after radiotherapy, and they remained at the same extremely low level even a year after radiotherapy. On the other hand, radiotherapy reduced the levels of CD8+ to 80% of pre-treatment values, and there was a trend towards the normalisation of their values a year after treatment completion, due to a faster effective doubling time of CD8+ cells, confirmed also in the study by Kuss et al. [29]. As a consequence, the CD4/CD8 index halved after radiotherapy and remained at the value of 1.1 1 year after radiotherapy. The ineffectiveness of the immune cell response mechanisms against tumour antigens may be in part due to the reduction in the number and activity of CD4+ T lymphocytes, also confirmed in our study, which impacts the function and activity of CD8+ T cells. CD4+ T cells play a key role in initiating and sustaining the CD8+ T lymphocyte-led immune responses directed against tumour cells. CD4+ T cells are important in preventing cytotoxic lymphocyte anergy, and are involved in the formation of the memory CD8+ subpopulation. They also stimulate macrophages and eosinophils present in the tumour stroma [33, 34].

Most striking was the effect of radiotherapy on naïve T lymphocytes (CD45 + RA+), whose levels plummeted to 20% of pre-treatment values in contrast to memory T lymphocytes (CD45 + RO+), whose levels merely halved after radiotherapy. Both values remained unchanged afterwards. Although the thymus was spared from radiation, probably due to partial thymus involution in elderly patients [35] and higher radiosensitivity of naïve cells [36], the reconstitution of T lymphocytes was significantly depressed. As noted before, even in the setting of exposing a small percentage of body surface to radiation therapy, when the irradiated area is rich in large blood vessels, this will cause prolonged immune alterations [12].

The immune defects of OSCC patients as well as other cancer patients are most likely due to a multitude of mechanisms, making the reversal of immune inhibition more difficult. Radiotherapy even worsens these defects, which could have a negative influence on the efficacy of RT itself and it might just be that reduced immunity induced by radiotherapy in selected group of patients within OSCC group makes them more susceptible to tumour recurrence and worse survival [12, 37].

Using biomarkers to stratify oral cancer patients’ risk of neck metastasis [38] is a rapidly developing field. We planned to search for possible markers to predict disease progression. We therefore divided patients into a treatment success and treatment failure group 2 years after radiotherapy, since recurrence most often appears within 2 years of treatment [39]. Although many authors have linked survival in head and neck cancer to many variables, such as CRP [21], lymphocyte/neutrophil ratio [40], monocytes [41] and thrombocytes [42] we did not find any correlation between pre-treatment values and disease progression or survival. The small sample size, which is the major limitation of our study, is probably the reason for this.

It should also be emphasized that the results of most of the cited publications regarding the head and neck region have dealt with a heterogeneous group of cancers, which are characterized by vastly different biological properties. Our study focused only on the immune changes in advanced squamous cell carcinoma of the oral cavity. Larger prospective studies are needed not only to confirm these findings, but also to address the possible underlying mechanisms that might link treatment-related lymphopenia to disease recurrence and survival.

## Conclusions

Our study has shown that surgery causes a profound inflammatory reaction and a fall in the levels of B lymphocytes as well as NK cells, while radiotherapy produces long lasting immune depression with levels of T, B lymphocytes, CD4+ T lymphocytes and CD4/CD8 index almost halved after 1 year. Even more striking was the influence on the naïve T lymphocytes that did not recover to levels above 20% of the starting values. None of the measured parameters allowed us to predict the clinical outcome before the commencement of treatment.

## Abbrevations

AOSCC: Advanced oral squamous cell carcinoma
CRP: C-reactive protein
ESR: Erythrocyte sedimentation rate
HNSCC: Head and neck squamous cell carcinoma
OSCC: Oral squamous cell cancers
RT: Radiotherapy
